# MDCFT-HSM: Modality-Shift-Aware RGB-T Small-Object Detection for Low-Altitude UAV Remote Sensing

**DOI:** 10.3390/s26165146

**Published:** 2026-08-14

**Authors:** Tianchen Long, Yanwen Wang, Hezhuo Yuan, Qian Zhang, Hongqing Ma, Rijin Zhou, Zhen Wang, Feng Wang

**Affiliations:** Xi’an Key Laboratory of Intelligent Perception and Autonomous Navigation for Low-Altitude Aircraft, School of Electronic Information, Xijing University, Xi’an 710123, China; 2408540106038@stu.xijing.edu.cn (T.L.);

**Keywords:** UAV remote sensing, RGB-T object detection, small-object detection, modality shift, controlled fine-tuning, feature recalibration

## Abstract

Low-altitude UAV RGB-T small-object detection is challenged by unequal modality reliability and the progressive attenuation of small-object evidence. To address these issues, this paper proposes Modality-Dominant Controlled Fine-Tuning with Hierarchical Small-Object Modeling (MDCFT-HSM), an ordered feature-flow framework for RGB-T detection. Based on dataset-level single-modality performance, MDCFT selects an initial protected branch and introduces auxiliary-modality information through zero-initialized residual mappings at the stem, P3, P4, and P5 stages, thereby limiting interference from degraded auxiliary features. Within this controlled feature flow, the High–Low Frequency Detail Enhancement module (HLFDE) preserves shallow boundary, texture, and local thermal-response cues; the Selective Boundary-Guided Aggregation module (SBGA) strengthens cross-level detail propagation in the neck; and the Multi-scale Guided Feature Recalibration module (MGFR) recalibrates the P3, P4, and P5 features before the Detect head. On RGBTDronePerson, MDCFT-HSM achieves 49.53% mAP@0.5 and 18.97% mAP@0.5:0.95 with 7.38 M parameters, 44.2 GFLOPs, and an inference speed of 60.0 FPS. On DroneVehicle, it achieves 83.60% mAP@0.5 and 63.28% mAP@0.5:0.95. Controlled robustness tests show limited tolerance to auxiliary RGB absence and mild cross-modal spatial misalignment, whereas severe IR degradation, IR absence, and larger spatial offsets cause substantial performance loss. These results demonstrate a competitive accuracy–complexity trade-off under a fixed dataset-level protected-branch configuration. The method does not provide online sample-level reliability adaptation or geometric registration.

## 1. Introduction

Small-object detection from unmanned aerial vehicle (UAV) visible–infrared (RGB-T) imagery serves as a fundamental visual task for low-altitude remote-sensing applications, including urban inspection, traffic surveillance, emergency rescue, and nighttime security monitoring. The task requires a model to respond jointly to the remote-sensing platform, sensor complementarity, and object scale. UAV imaging introduces larger viewpoint changes, smaller object pixel footprints, and stronger background-structure interference. Paired RGB and IR inputs further require the model to determine when the two sensor cues are complementary and when they interfere with each other. RGBTDronePerson evaluates person, rider, and crowd categories under UAV RGB-T imaging and reveals the joint difficulty caused by tiny objects, dense distributions, and complex backgrounds [1]. This paper, therefore, studies RGB-T small-object detection from the perspective of low-altitude UAV remote sensing, with an emphasis on how sensor reliability differences and small-scale evidence attenuation affect the detection feature flow. Multi-rotor UAV platforms have also been reviewed as flexible platforms for meteorological sensing and low-altitude environmental observation [2].

**Figure 1:**
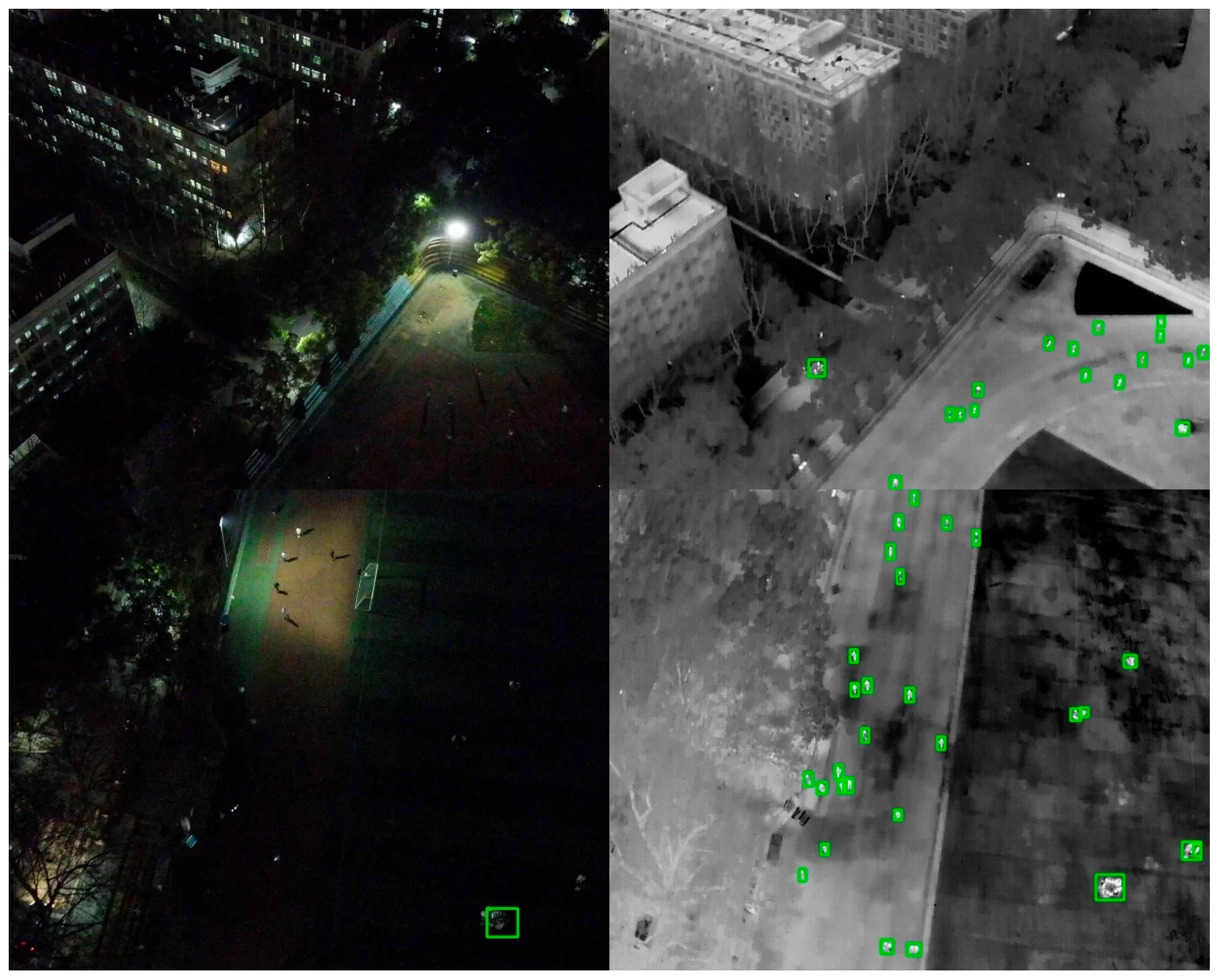
Modality degradation, modality shift, and weak small-object evidence in UAV RGB-T detection. Green boxes indicate annotated small-object instances in the infrared image.

In this paper, modality shift refers to changes in the relative reliability of RGB and IR evidence caused by modality-specific degradation or modality absence. Cross-modal spatial misalignment is treated separately as a geometric inconsistency between the paired inputs. Such reliability variations can be obscured by simple RGB-T fusion. RGB images provide color, texture, and geometric boundary cues, but they can be submerged by background responses at night or under shadow, smoke, low contrast, or motion blur. Infrared images highlight thermal objects, yet they may suffer from sensor noise, thermal-background diffusion, local saturation, and insufficient texture [3]. Weak alignment, cross-modal conflict, and modality imbalance further show that cross-modal information is not always equivalently complementary. Unconditional concatenation, averaging, or symmetric attention exchange may propagate local false responses into later feature layers [3]. In UAV small-object scenes, this risk is amplified by object size because a small number of incorrect boundaries or background hot spots can become high-level semantic responses after downsampling. The fusion process should, therefore, protect the initially selected representation before absorbing useful evidence from the auxiliary sensor in a controlled manner. Figure 1 illustrates common modality degradation, modality shift, and weakened small-object evidence in low-altitude RGB-T detection.

Small-object evidence attenuation is another difficulty coupled with modality shift. In UAV images, objects often occupy only a few pixels. Boundary, texture, local thermal response, and neighborhood context still exist in shallow layers, but they are easily diluted by large background structures after repeated downsampling [1]. Adding attention only to high-level semantics or the Detect head is insufficient when boundary evidence has already been weakened in shallow and middle feature stages. Shallow sharpening alone may also treat background texture as object evidence. Frequency enhancement and high- and low-frequency decomposition suggest that high-frequency residuals help retain boundaries, textures, and local thermal responses, whereas a low-frequency structure stabilizes the object–background context. Both should work together with channel selection, residual fusion, and later semantic aggregation [4]. UAV RGB-T small-object detection, therefore, requires an ordered feature flow that controls the entry of auxiliary-modality features, preserves high- and low-frequency details at shallow layers, transfers small-object spatial cues through the neck, and recalibrates multi-scale features before the Detect head.

CFT [5], YOLOFusion [6], ICAFusion [7], and DPDETR [8] use Transformer modeling, cross-modal attention, iterative cross-attention, and position decoupling, respectively, to improve modality interaction.

QFDet [1] addresses tiny-target detection, while PTANet [9], VTsARNet [10], AODet [11], SLBAF-Net [12], DEYOLO [13], and DEGF-YOLO [14] introduce target awareness, Transformer representations, anti-occlusion design, lightweight fusion, dual-feature enhancement, and dynamic gating into UAV RGB-T scenes.

DHANet [15], multibranch progressive fusion [16], and prior-enhanced mixture-of-experts fusion [17] further indicate that multimodal remote-sensing detection should handle hierarchical information and modality reliability in stages.

We propose MDCFT-HSM, a modality-shift-aware feature-flow framework for low-altitude UAV RGB-T small-object detection. The contributions are threefold. First, we formulate UAV RGB-T small-object detection as a coupled problem of unequal modality reliability and progressive small-object evidence attenuation. MDCFT uses a dataset-level modality prior to selecting an initial protected branch and controls auxiliary-feature entry through zero-initialized residual injection. Second, we organize HLFDE, SBGA, and MGFR into an ordered feature flow that preserves shallow details, transfers low-level spatial evidence through the neck, and recalibrates multi-scale features before the Detect head. The principal contribution lies in the task-oriented ordering and controlled interaction of these components rather than in claiming that each underlying operation is individually new. Third, we evaluate the proposed design through component ablation, protected-branch and injection-location studies, module replacements, modality-degradation and spatial-misalignment tests, scale-stratified and condition-stratified evaluation, cross-method comparison, and an additional experiment on DroneVehicle.

## 2. Related Work

### 2.1. RGB-T UAV and Remote Sensing Object Detection

RGB-T UAV detection studies share the goal of converting visible-texture cues and thermal–infrared responses into reliable remote-sensing object evidence from low-altitude views. RGBTDronePerson includes person, rider, and crowd categories in paired UAV RGB-T evaluation and reveals the common challenges caused by tiny objects, dense distributions, and complex backgrounds [1]. PTANet [9] introduces progressive target awareness for drone-based RGB-T person detection, whereas VTsARNet [10] strengthens person detection in paired RGB-T aerial images with Transformer representations. These studies show that UAV RGB-T detection must jointly handle aerial viewpoint, object scale, background interference, and modality-quality variation.

Multispectral and RGB-T object detection methods provide the main technical background for this work. CFT [5] uses a cross-modality Transformer to model multispectral feature relations. YOLOFusion/CMAFF [6] models common and differential information through cross-modal attention. ICAFusion [7] iteratively refines fusion through cross-attention, and DPDETR [8] reduces infrared–visible response differences through position decoupling. Compared with general RGB-T detectors, UAV RGB-T small-object scenes are more sensitive to local boundaries, thermal responses, and category density. Cross-modal interaction should, therefore, be discussed together with small-object evidence preservation.

DHANet [15] uses dual-stream hierarchical interaction for multimodal drone detection. Multibranch progressive fusion [16] organizes RGB-T aerial detection as staged feature fusion, and prior-enhanced mixture-of-experts fusion [17] introduces reliability priors and expert routing into multimodal remote-sensing object detection.

QFDet [1], AODet [11], SLBAF-Net [12], DEYOLO [13], and DEGF-YOLO [14] improve UAV RGB-T detection from the perspectives of tiny-target quality awareness, occlusion robustness, lightweight design, dual-feature enhancement, and dynamic gating. LF-DETR [18] provides a recent reference for evaluating aerial RGB-infrared pedestrian detection on RGBTDronePerson, including class-wise AP, mAP@0.5, FPS, parameters, and GFLOPs.

Recent studies have also reconsidered RGB-T fusion from the perspective of modality-specific representation. RSDet [19] adopts a removal-then-selection strategy for coarse-to-fine RGB-T fusion, whereas M^2^D-LIF [20] emphasizes mono-modality feature learning within multimodal detection. These methods differ from MDCFT-HSM, which protects a dataset-level selected branch through zero-initialized residual injection and then performs staged small-object feature modeling.

### 2.2. Small-Object Representation and Detail Enhancement

The core difficulty of small-object detection lies in scarce object evidence, easily lost boundaries, and context that can be occupied by background structures. Many objects in RGBTDronePerson are very small. Although vehicle categories in DroneVehicle are relatively larger, local occlusion and scale variation may still occur under high-altitude views and complex road backgrounds [1]. A detector must retain sufficient boundary and texture cues at shallow layers while forming stable semantic responses at high layers. Task-specific vehicle detectors have likewise been developed for intelligent-factory scenes with partial occlusion and background interference [21].

HDNet [4] enhances infrared small-object representations using multi-scale high-frequency information, and FFE-DETR [22] uses frequency-aware feature enhancement to alleviate foreground-boundary blurring and contrast degradation at low-light detection. Related small- and medium-object detection work has also studied dense ship scenes using improved SSD-based feature fusion [23].

High-and low-frequency decomposition further suggests that detail enhancement should not focus only on high-frequency residuals. High-frequency components can describe boundaries, textures, and local thermal responses, whereas low-frequency components preserve structural contours and background context. If only high-frequency information is enhanced, the model may treat background texture as object evidence. If only low-frequency information is used, fine boundaries may be smoothed [24]. HLFDE follows this idea at the shallow stage. It obtains low-frequency structure by average pooling, separates high-frequency residuals by subtracting the upsampled low-frequency component from the original feature, and then performs residual fusion through two branches and channel attention. This design serves detection features rather than directly transferring an image-restoration conclusion to detection.

Existing small-object and frequency-enhancement methods mainly focus on local details, multi-scale features, or low-light enhancement. This paper adds shallow-detail preservation to the RGB-T feature-flow repair chain, forming an ablatable staged relation with the controlled injection of MDCFT, the cross-level evidence transfer of SBGA, and the pre-prediction recalibration of MGFR. In other words, HLFDE is not an isolated sharpening module. It provides clearer small-object evidence for the later neck and detection features.

### 2.3. Modality-Shift-Aware Fusion and Feature Recalibration

Weakly aligned feature fusion [3] points out that multimodal image pairs may have positional offsets and response differences. Cross-modal conflict-aware learning [25] emphasizes that different modalities may provide contradictory target and background cues. Effectiveness-guided information sharing [26] further shows that cross-modal exchange should be regulated by modality effectiveness rather than unconditional sharing.

A comprehensive review of multimodal remote-sensing data fusion indicates that cross-sensor complementarity should be judged according to sensor differences, scene conditions, and task scale [27]. A survey of RGB-T vision tasks also emphasizes that differences between visible textures and thermal–infrared saliency affect target localization and background suppression [28].

Research on modality imbalance provides a direct background for dataset-level protected-branch selection. Visible and thermal modalities in multispectral pedestrian detection are not stable across environments, and the advantage of one modality may vary with the dataset, time, weather, and sensor quality [29]. Therefore, we do not assume that IR or RGB is naturally the dominant modality. The protected branch is selected using single-modality experiments or sensor-quality priors. In the reported RGBTDronePerson setting, IR-only clearly outperforms RGB-only, so the reported configuration uses infrared as the protected feature flow and RGB as the auxiliary sensor evidence.

Feature recalibration studies focus on multi-scale prediction features before the Detect head. YOLO-MFG [30] uses gated attention and multi-scale feature preservation for remote-sensing object detection, indicating that feature selection before the Detect head directly affects the final box and class outputs. Unlike approaches that add interaction only in the backbone or neck, MGFR is placed before the Detect head on P3, P4, and P5. Its purpose is to combine local context and channel-compressed spatial saliency before final prediction and perform dual-path selection and cross-modulation between the current-scale feature and the guidance feature.

Cross-level aggregation studies are also related to our design. The dual-aggregation idea in DuAT shows that low-level spatial details and high-level semantics should be selectively coordinated before aggregation, rather than directly concatenated [31]. We adapt this boundary-guided aggregation idea to the YOLO neck and formulate SBGA for UAV RGB-T small-object detection. SBGA performs independent channel projection, spatial alignment, and bidirectional Sigmoid-gated compensation between low-level details and high-level semantic responses. Overall, the existing methods have covered weak alignment, conflict awareness, effectiveness-guided sharing, gated attention, and cross-level aggregation. This work organizes these inspirations into a feature-flow chain for modality shift and small-object evidence attenuation and verifies the role of each stage through unified ablation.

These studies point to a clear design requirement: cross-modal fusion should control information entry according to the reliability difference between RGB and IR, and small-object detection should continuously preserve usable evidence at the shallow layer, the neck, and before the Detect head. MDCFT-HSM implements this requirement as a continuous feature flow, where MDCFT, HLFDE, SBGA, and MGFR correspond to different stages of modality-shift control and small-object evidence preservation.

## 3. Method

### 3.1. Overall Architecture

The overall goal of MDCFT-HSM is to build a stable and ablatable UAV small-object feature flow for paired RGB and IR inputs. The design is driven by the coupled failure of modality shift and small-object evidence attenuation. MDCFT handles protected-branch preservation and controlled auxiliary-modality injection. HLFDE, SBGA, and MGFR then preserve and transfer small-object evidence at the shallow stage, in the neck, and before the Detect head, respectively. Equation (1) first defines the input so that all subsequent features are tied to the same paired sensor observation.

(1) $$X=Concat(X_{rgb},X_{ir})\;\in R^{H\times W\times 6}{(X}_{rgb},X_{ir}\in R^{H\times W\times 3})$$

In Equation (1), Xrgb and Xir denote the visible and infrared inputs in the paired sample. Both are represented as H × W × 3 tensors for input organization. The concatenated X denotes the paired six-channel data package used in the training pipeline; it does not indicate early fusion in the feature extractor. In implementation, X is split into RGB and IR branches before feature extraction, and cross-modal reliability is handled by protected-branch preservation and zero-initialized residual injection in MDCFT. Equations (2) and (3) then define the complete feature flow from controlled cross-modal entry to feature recalibration before the Detect head.

P = {P_3_, P_4_, P_5_} = N_SBGA_(HLFDE(B_MDCFT_(X))) (2)

In Equation (2), BMDCFT denotes modality-dominant controlled feature extraction after the six-channel package X is split into RGB and IR branches. HLFDE denotes shallow high- and low-frequency detail enhancement, and NSBGA denotes SBGA-based neck aggregation. The resulting feature set P = {P3, P4, P5} retains progressively different spatial-semantic levels: P3 keeps more small-object detail, whereas P4 and P5 carry stronger semantics.

(3) $$Y=P^{*}=MGFR\left(P\right)$$

**Figure 2:**
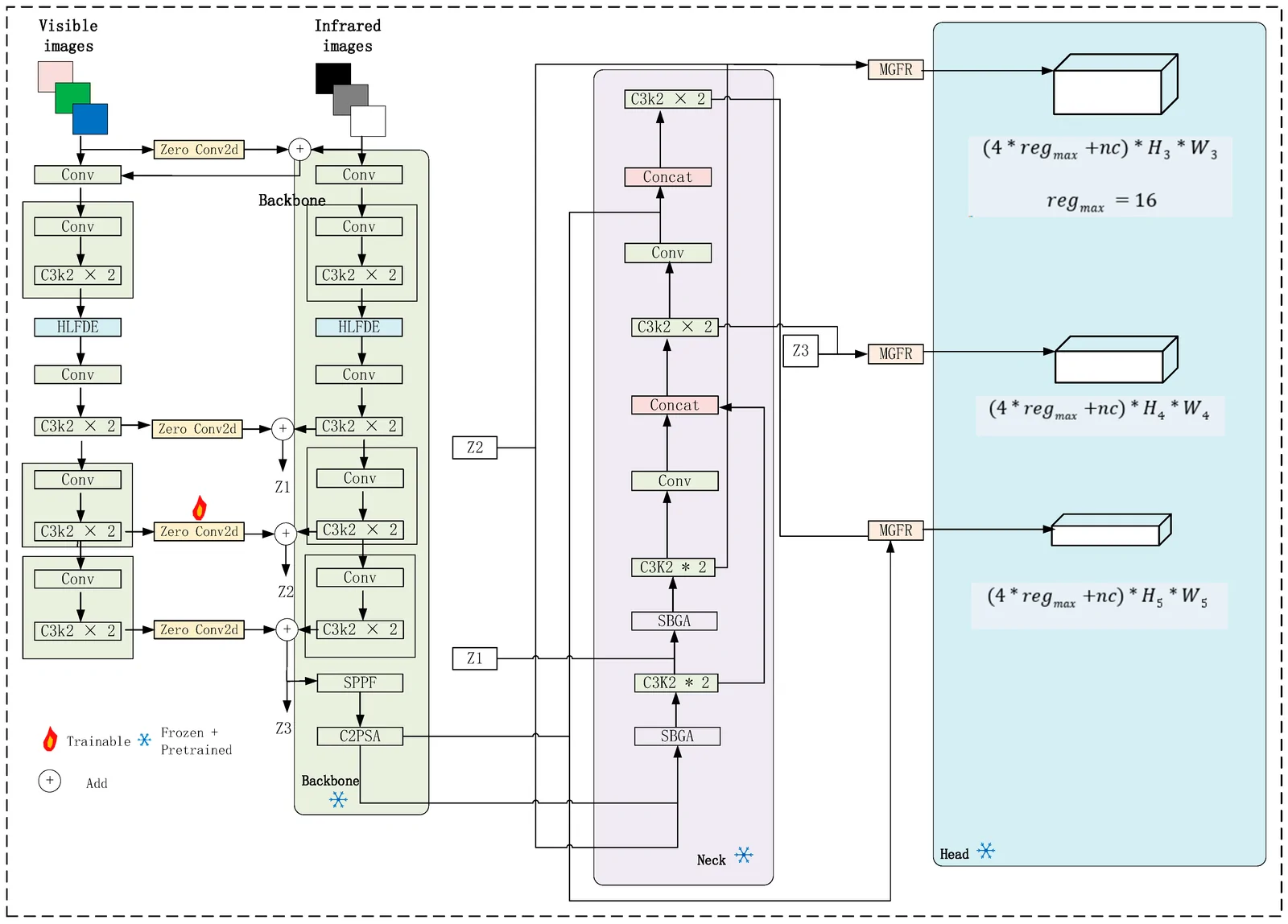
Overall architecture of MDCFT-HSM. The infrared branch is used as the protected feature flow in the reported RGBTDronePerson setting, while RGB features are injected through zero-initialized residual paths. HLFDE, SBGA, and MGFR are placed at the shallow stage, neck aggregation stage, and before the detection head, respectively.

In Equation (3), MGFR recalibrates P3, P4, and P5 before the standard Detect head D, which outputs class scores, bounding boxes, and confidence scores. The overall architecture is shown in Figure 2, and the training objective is defined in Section 3.6.

### 3.2. MDCFT

MDCFT is designed to reduce the interference caused by unequal modality reliability. In the reported RGBTDronePerson setting, the IR branch is selected as the initial protected branch because the IR-only baseline outperforms the RGB-only baseline. The pretrained IR branch is frozen, while the RGB branch supplies trainable auxiliary features through zero-initialized residual mappings at the stem, P3, P4, and P5 stages. Unlike direct addition, concatenation, or symmetric feature exchange, the auxiliary modality initially has no influence on the protected representation.

(4) $$F_{k}^{m}=M_{k}(X_{m}),\;m\;\in \;\{rgb,\;ir\}$$

Let m and a denote the protected and auxiliary modalities, respectively. $F_{k}^{m}$ and $F_{k}^{a}$ are their features at stage k ∈ {stem, P3, P4, P5}. The protected feature is generated by the frozen branch, whereas the auxiliary feature is produced by the trainable auxiliary branch at the corresponding scale.

(5) $$F_{k}^{a}=A_{k}(X_{a}),\;a\;\neq \;m$$

(6) $$\Delta_{k}=Z_{k}\left(F_{k}^{a}\right)=W_{k}^{\left(0\right)}\;*\;F_{k}^{a}+b_{k}^{\left(0\right)},\;W_{k}^{\left(0\right)}=0,\;b_{k}^{\left(0\right)}=0$$

Here, * denotes the convolution operation.

(7) $$\;{\tilde{F}}_{k}=F_{k}^{m}+\Delta_{k}$$

(8) $$\mathrm{At}\;t=0:\;\Delta_{k}=0,\;{\tilde{F}}_{k}=F_{k}^{m}$$

At each injection stage, the zero-initialized mapping $Z_{k}$ generates the auxiliary residual $\Delta_{k}$ from $F_{k}^{a}$. Because the weights and biases are initialized to zero, $\Delta_{k}$ = 0 at the beginning of training, and the fused feature initially equals the protected feature $F_{k}^{m}$. During optimization, $Z_{k}$ progressively learns the auxiliary correction required by the detection objective. MDCFT controls the initial influence of the auxiliary modality but does not perform geometric registration or online reliability estimation.

**Figure 3:**
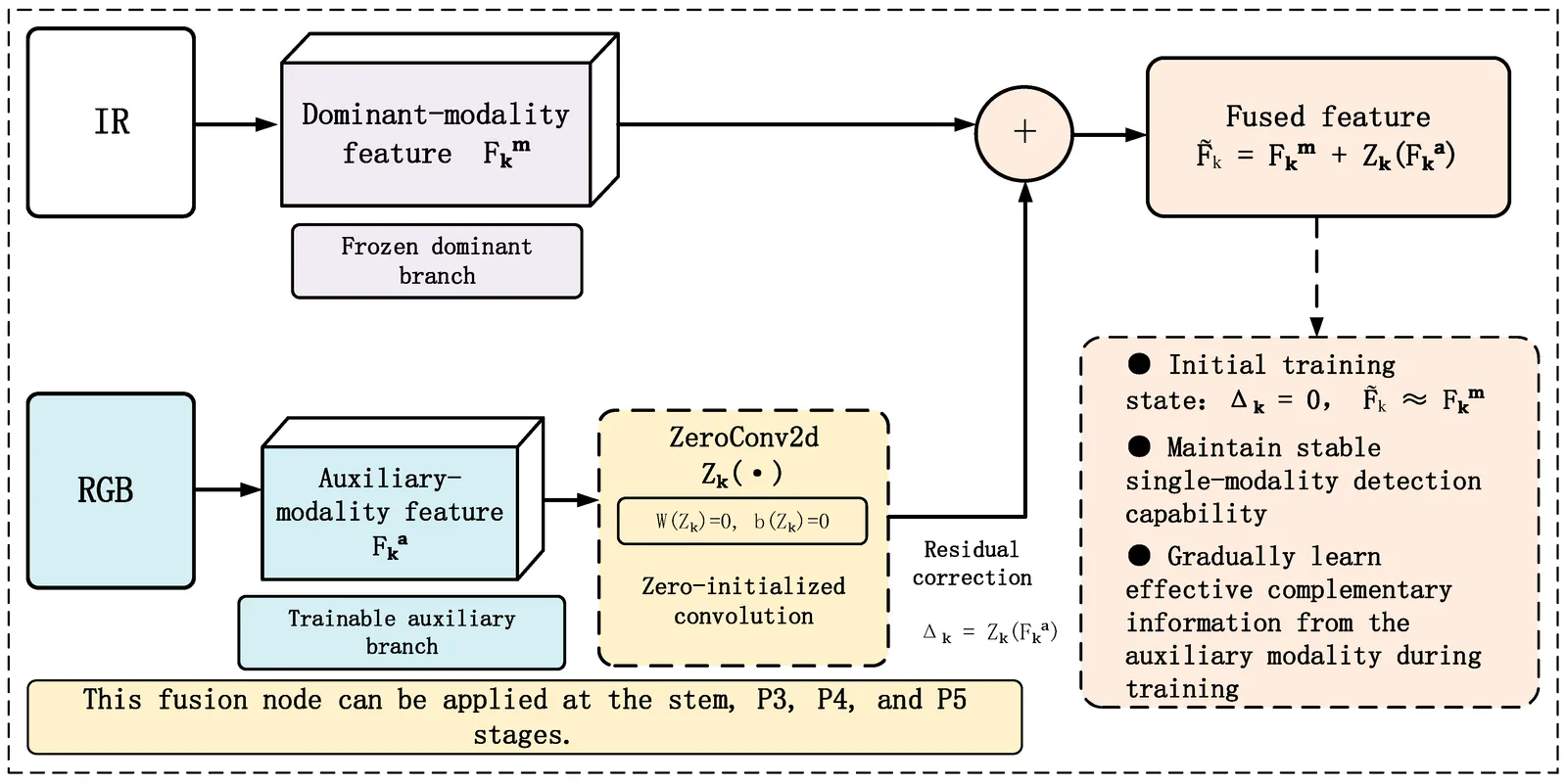
MDCFT with a protected branch and zero-initialized residual injection. The residual injection node is applied at the stem, P3, P4, and P5 stages.

In the reported configuration, the pretrained IR backbone, neck, and detection head are frozen. The RGB auxiliary branch, zero-initialized residual mappings, HLFDE, SBGA, and MGFR remain trainable. The MDCFT protected-branch and zero-initialized residual-injection mechanism is illustrated in Figure 3.

### 3.3. HLFDE

HLFDE addresses the shallow-detail attenuation caused by very small objects. UAV pedestrians, riders, and crowds often occupy only a small number of pixels. Boundaries, textures, and local thermal responses are still visible at the shallow P2/4 stage, but they are easily diluted by background structures after continuous downsampling [24]. HLFDE is, therefore, placed where shallow details are still sufficiently available, and it separates low-frequency structure from high-frequency residuals to protect small-object evidence.

(9) $$F_{low}=\mathrm{AvgPool}\left(F\right)$$

(10) $$F_{low}^{↑}=\mathrm{Up}\left(F_{low}\right)$$

(11) $$F_{high}=F-F_{low}^{↑}$$

Given the shallow feature F, average pooling followed by interpolation produces the aligned low-frequency component $F_{low}$. The high-frequency residual is obtained as $F_{high}$ = F − $F_{low}$. This decomposition separates broader structural information from local boundary, texture, and thermal-response cues. The subtraction isolates residual high-frequency responses from the original shallow feature.

(12) $$E_{high}=\mathrm{Dense}\left(F_{high}\right)$$

(13) $$E_{low}=\mathrm{Up}\left(U\left(F_{low}\right)\right)$$

(14) $$F_{HLFDE}=F+C_{2}\left(A\left(C_{1}\left(\left[E_{high},E_{low}\right]\right)\right)\right)$$

**Figure 4:**
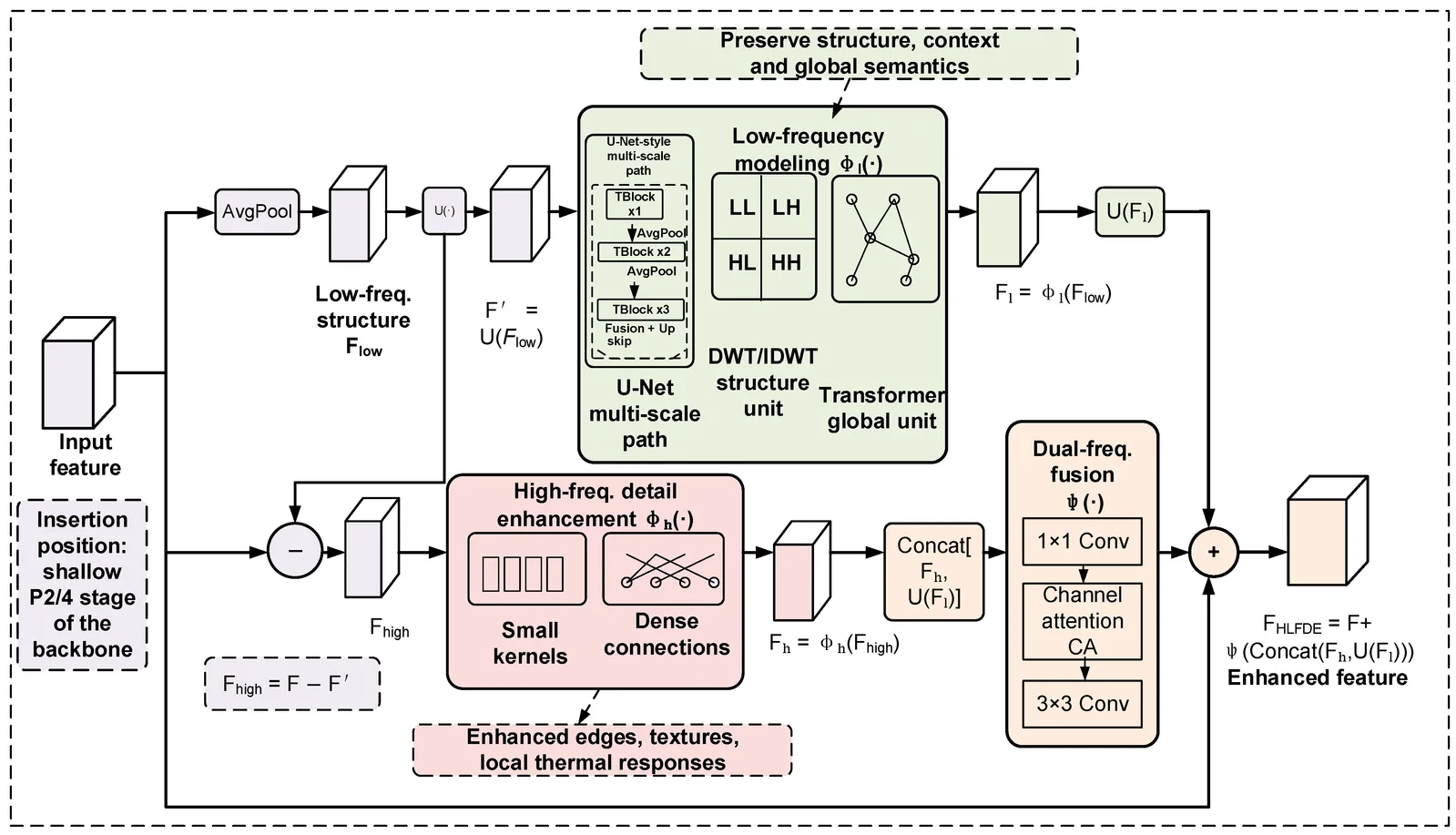
HLFDE for shallow high- and low-frequency detail enhancement. The low-frequency branch preserves structural context, whereas the high-frequency residual branch enhances boundaries, textures, and local thermal responses.

The high-frequency branch uses dense local convolution to enhance boundaries and local thermal responses. The low-frequency branch uses a U-Net-style path with Transformer and DWT/IDWT units to model the broader structural context. Their outputs are fused by channel attention and convolution and are then added residually to the original shallow feature. No auxiliary frequency-domain loss is used. The HLFDE structure is shown in Figure 4.

### 3.4. SBGA

SBGA targets the stage where small-object evidence is easily diluted during cross-level aggregation in the neck. Low-level features retain more spatial boundaries of small objects, but they also contain background texture and noise. High-level features have stronger semantics, but the locations and fine boundaries of small objects may become coarse. Direct concatenation can, therefore, dilute small-object details during cross-level aggregation. SBGA performs bidirectional gated compensation before concatenation, allowing shallow small-object details to enter the neck features more selectively. Equation (15) first defines the two input projections.

(15) $$L=C_{l}\left(F_{l}\right),\;H=C_{h}(F_{h})$$

SBGA receives a low-level detail feature and a high-level semantic feature. Independent 1 × 1 projections align their channel dimensions, and the high-level feature is resized to the spatial resolution of the low-level feature.

(16) R(X; T): X → T scale, T ∈ {L, H}

(17) $$G_{H}=\sigma (H),\;G_{L}=\sigma (L)$$

Independent Sigmoid gates perform bidirectional compensation. High-level semantics selectively refine low-level details, whereas the updated low-level evidence constrains high-level responses. These gates are independent modulation maps rather than Softmax path-selection probabilities.

(18) $$L^{\prime }=L+L\;⊙\;G_{L}+(1-G_{L})\;⊙\;R(G_{H}\;⊙\;H;\;L)$$

(19) $$H^{\prime }=H+H\;⊙\;G_{H}+(1-G_{H})\;⊙\;R(G_{L}\;⊙\;L^{\prime };\;H)$$

(20) $$F_{SBGA}=C_{3\times 3}(Concat[R(H^{\prime };\;L),\;L^{\prime }])$$

**Figure 5:**
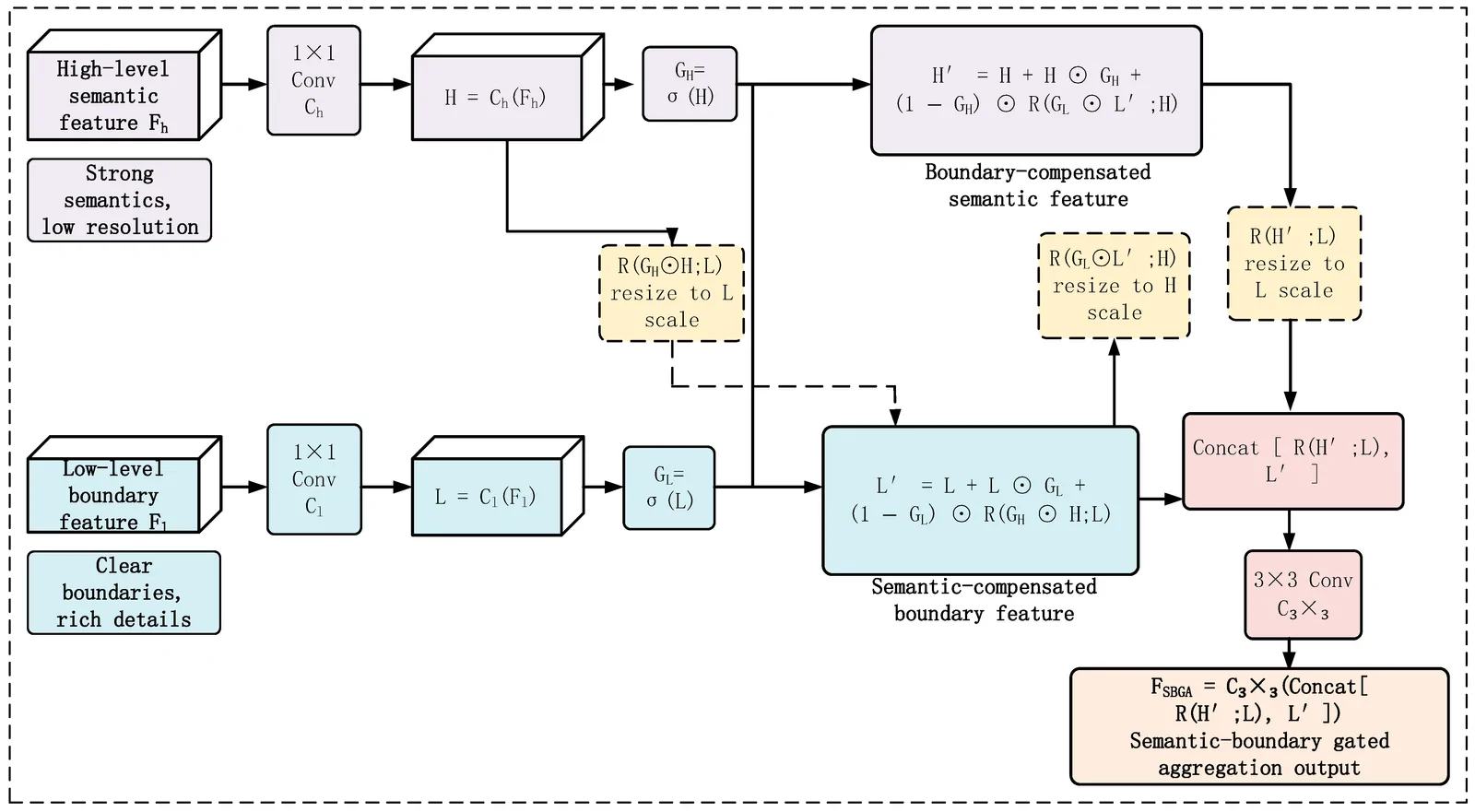
SBGA for gated cross-level aggregation in the neck. Low-level boundary cues and high-level semantic responses are compensated through independent Sigmoid gates before concatenation.

After compensation, the high-level feature is resized to the low-level resolution, concatenated with the updated low-level feature, and fused by a 3 × 3 convolution to produce $F_{SBGA}$. The SBGA structure is shown in Figure 5.

### 3.5. MGFR

MGFR addresses the stage where multi-scale small-object evidence before the Detect head can still be diluted by background responses and scale differences. Even after MDCFT controls modality shift, HLFDE preserves shallow details, and SBGA helps small-object evidence pass through the neck, the final P3, P4, and P5 detection features may still be disturbed by object-scale differences, background hot spots, and local texture. MGFR, therefore, serves as the final recalibration step before the Detect head, performing local-context and spatial-saliency-guided selection with cross-modulation for multi-scale small-object evidence.

P = φ_local_(x),  Q = φ_saliency_(g),  R = BN(P + Q) (21)

At each detection scale, MGFR receives the current feature x and a guidance feature g. A local branch processes x, while a spatial-saliency branch summarizes the channel-wise average and maximum responses of g. The fused representation is then used to generate two path-selection weights.

(22) [α, β]=Softmax(S(R))

A 1 × 1 convolution followed by a two-path Softmax produces the weights α and β. Softmax determines the relative contribution of the current-scale and guidance features, whereas the Sigmoid gates control the subsequent cross-modulation strength.

(23) $$x^{\prime }=x+\alpha \;⊙\;x,\;g^{\prime }=g+\beta \;⊙\;g,\;I=\left[\sigma \left(g^{\prime }\right)\;⊙\;x^{\prime }\right]\;⊙\;\left[\sigma \left(x^{\prime }\right)\;⊙\;g^{\prime }\right]$$

(24) $$M=\sigma \left({BN}_{1}\left(W\left(I\right)\right)\right),\;F_{MGFR}={BN}_{2}\left(C\left(M\;⊙\;x\right)\right)$$

**Figure 6:**
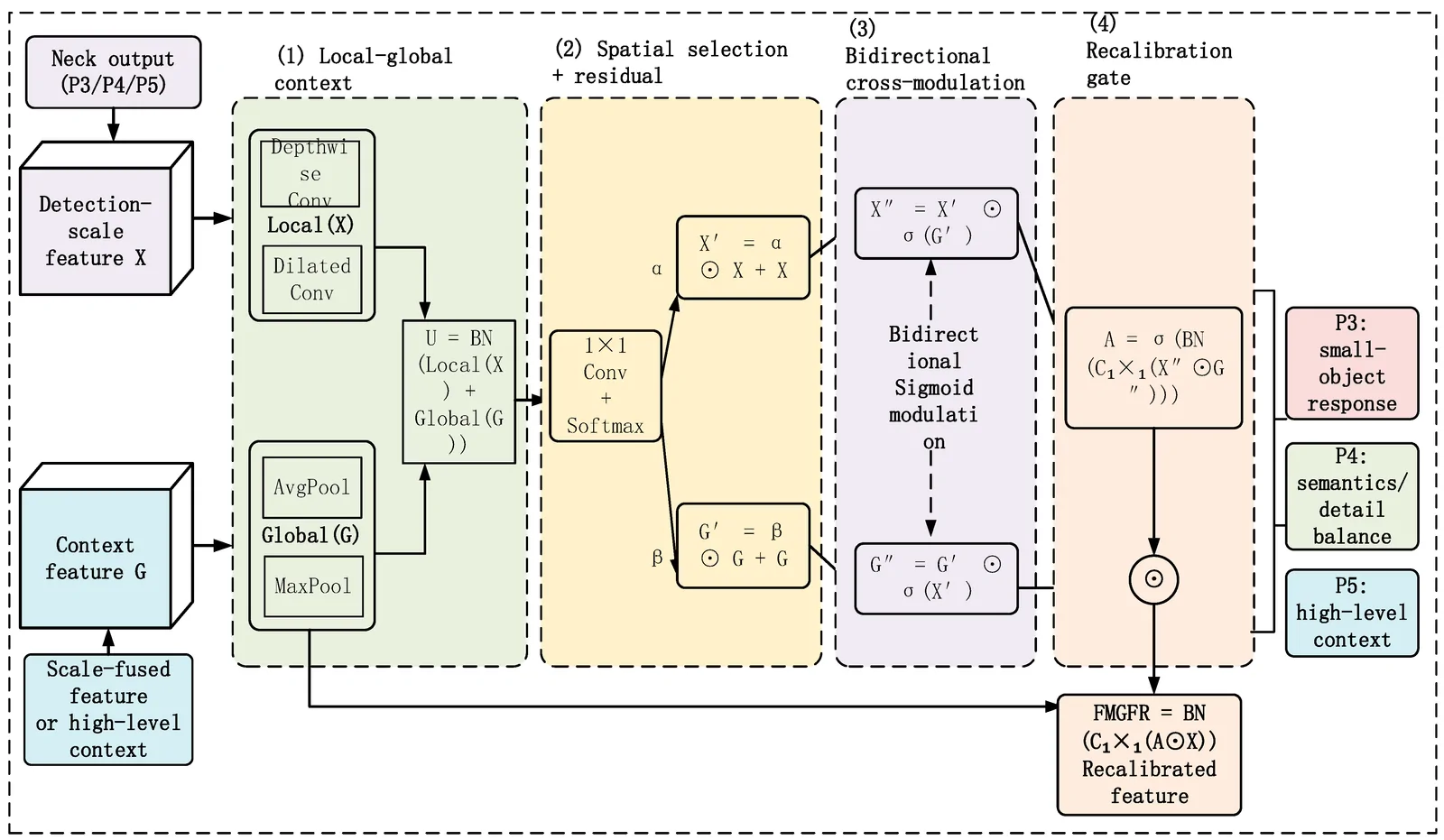
MGFR for feature recalibration before the Detect head. The module combines local context and spatial-saliency guidance, performs Softmax-based path selection, and applies bidirectional Sigmoid cross-modulation to the P3, P4, and P5 features.

The weighted features x′ and g′ modulate each other through Sigmoid gates. Their interaction generates a recalibration map M, which reweights the original feature x before the final convolutional output. Softmax is used for relative path selection, whereas the Sigmoid controls the cross-modulation strength. The MGFR structure is shown in Figure 6. 

### 3.6. Training Objective and Implementation Details

MDCFT-HSM does not introduce additional supervised losses. The training objective follows the original detector losses for box regression, classification, and DFL. This choice ensures that ablation differences mainly correspond to the feature-flow design of MDCFT, HLFDE, SBGA, and MGFR, rather than changes in the loss function. Equation (25) gives the training objective.

(25) $$L\;=\lambda_{box}L_{box}+\lambda_{cls}L_{cls}+\lambda_{dfl}L_{dfl}$$

In Equation (25), the box term denotes the bounding-box regression loss, the class term denotes the classification loss, and the DFL term denotes the distribution focal loss. The lambda weights denote the weights of the loss terms and do not introduce additional labels or auxiliary supervision.

The input size is 640 × 640. The paired RGB and IR data are packaged as a six-channel input and then split into two modality branches. Each single-channel IR image is replicated across three channels before being packaged with the RGB image.

The experiments were conducted on a workstation equipped with an Intel Core i9-13900K CPU (Intel Corporation, Santa Clara, CA, USA), 32 GB of system memory, and an NVIDIA GeForce RTX 4090 GPU with 24 GB of video memory (NVIDIA Corporation, Santa Clara, CA, USA). The software environment consisted of Python 3.8, PyTorch 1.13, CUDA 11.7, cuDNN 8.5, and Ultralytics 8.3.75. The experiments used 150 training epochs. The paired input was organized as six channels, including three RGB channels and three replicated IR channels. The protected IR branch was initialized from the trained IR-only checkpoint, whereas the auxiliary RGB branch was initialized from YOLO11n pretrained weights. When a three-channel input was required, the single-channel IR image was replicated using Gray2BGR. HLFDE used one-level Haar DWT/IDWT, two Transformer blocks per stage, and feature-channel dimensions of 64, 128, and 256 at P3, P4, and P5, respectively. The pretrained IR backbone, neck, and Detect head were frozen. The RGB auxiliary branch, zero-initialized residual mappings, HLFDE, SBGA, and MGFR remained trainable. The operating-system version was not preserved in the archived experimental record and is, therefore, not inferred.

**Figure 7:**
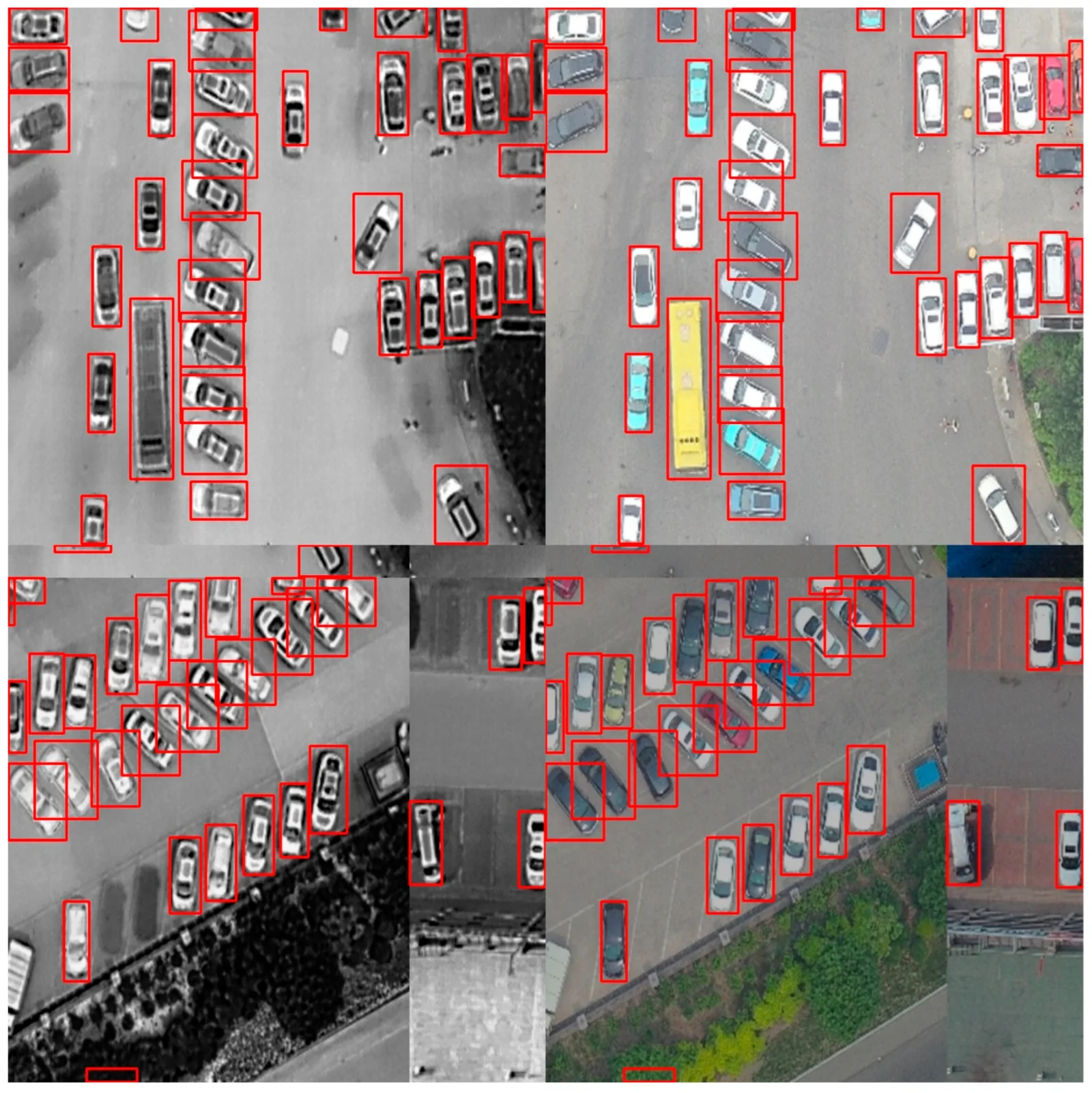
Paired RGB-T samples with aligned bounding boxes were used for geometrically consistent augmentation.

Paired RGB and IR augmentation followed the principle of geometric consistency. The same geometric transformation parameters were applied to the RGB image, IR image, and bounding boxes during random scaling, cropping, flipping, and translation. Modality-specific intensity perturbations were applied independently without changing the spatial correspondence of the paired inputs. This procedure avoided artificial cross-modal spatial misalignment during training, ensuring that the auxiliary residual learned by MDCFT was derived from paired complementary information rather than augmentation-induced geometric differences. Figure 7 shows paired RGB-T samples with aligned bounding boxes.

## 4. Experiments

### 4.1. Datasets and Experimental Setup

The experiments use RGBTDronePerson as the main dataset and DroneVehicle as an additional vehicle-oriented UAV RGB-T scenario. RGBTDronePerson is designed for UAV RGB-T pedestrian detection and includes person, rider, and crowd categories, as well as small objects, dense distributions, and complex backgrounds [1]. DroneVehicle is designed for UAV RGB-T vehicle detection, and HyperDet provides a systematic description of the paired-image scale, vehicle categories, evaluation metrics, and comparison settings of this dataset [32]. These datasets support evaluation in pedestrian-oriented and vehicle-oriented UAV RGB-T scenarios.

The experiments are organized under the evaluation setting adopted in this work. Unreported items are retained as dashes in the tables. The RGBTDronePerson comparison table uses Crowd, Person, Rider, mAP@0.5, FPS, Params, and GFLOPs. The DroneVehicle table uses per-class AP, mAP@0.5, mAP@0.5:0.95, Params, GFLOPs, and FPS. Because FPS is sensitive to implementation details, such as the hardware platform, inference backend, input resolution, post-processing, and batch setting, Params and GFLOPs are reported together to interpret accuracy, model size, and computational cost. The complete training and evaluation configuration is summarized in Table 1.

Inference latency was measured using a 640 × 640 input resolution, a batch size of 1, FP32 precision, 100 warm-up iterations, and 1000 synchronized timing iterations. Each timed interval included model forward propagation, output decoding, and NMS with confidence and IoU thresholds of 0.25 and 0.7, respectively, while excluding disk I/O. CUDA synchronization was performed immediately before and after each timed interval.

### 4.2. Ablation Study

The ablation study evaluates the contributions of MDCFT, HLFDE, SBGA, and MGFR in stages. MDCFT controls auxiliary-feature injection under unequal modality reliability. HLFDE preserves shallow details. SBGA transfers low-level information across the neck stages, and MGFR recalibrates the features before the Detect head. The corresponding ablation results are summarized in Table 2.

YOLOv11n (IR-only) reaches 44.19% mAP@0.5, whereas YOLOv11n (RGB-only) reaches 5.95%, indicating that IR provides the stronger single-modality initialization on this dataset. The two baselines use the same dataset split, detector architecture, optimization schedule, augmentation protocol, and evaluation procedure; only the input modality differs. The RGB-only model shows normal convergence, and the measured result is, therefore, retained. MDCFT (IR-protected) reaches 44.38%, remaining close to the IR-only baseline. This result indicates that controlled residual injection preserves the protected feature flow and provides a stable starting point for the subsequent modules.

Module increments should be interpreted along the small-object evidence chain. After HLFDE is added, mAP@0.5 reaches 47.37%, indicating preservation of shallow high- and low-frequency details. HLFDE with SBGA reaches 48.17%, showing that gated transfer of low-level details in the neck further helps preserve small-object evidence. MGFR alone improves MDCFT (IR-protected) from 44.38% to 46.00%, indicating that feature recalibration before the Detect head is useful. With HLFDE, SBGA, and MGFR used together, the complete model reaches 0.5516 Precision, 0.5226 Recall, 49.53% mAP@0.5, 44.2 GFLOPs, and a 14.6 MB weight size. This indicates that the benefit comes from complementary modules organized around the small-object evidence chain.

The module combinations are not linearly additive. MGFR is most effective when HLFDE and SBGA have already refined the preceding features. The protected branch is selected from dataset-level single-modality evidence and should be re-evaluated under different sensor settings.

### 4.3. Design-Choice and Robustness Analysis

This section examines MDCFT design choices, modality degradation and spatial misalignment, and scale- and condition-stratified performance under a common experimental setup.

#### 4.3.1. MDCFT Design Choices and Module Replacements

Table 3 compares the protected-branch direction at the MDCFT-only stage. The RGB-protected MDCFT configuration achieves 45.896% mAP@0.5, exceeding the 44.380% obtained with the IR-protected configuration, although the RGB-only baseline is substantially weaker than the IR-only baseline. This result shows that single-modality performance provides only a dataset-level initialization prior and cannot reliably determine the optimal multimodal feature-flow direction. The protected branch should therefore be regarded as a heuristic design choice that must be re-evaluated across different datasets and sensor conditions. The subsequent HLFDE, SBGA, and MGFR experiments retained the IR-protected direction to preserve a controlled module-ablation sequence. Switching the protected direction after observing the MDCFT-only direction-sensitivity result would introduce post-hoc direction selection and break the consistency of the controlled ablation sequence. Therefore, the RGB-protected result is reported only as a direction-sensitivity analysis rather than as a second complete MDCFT-HSM configuration. The complete MDCFT-HSM was not evaluated under the RGB-protected configuration in the current revision.

All design-choice experiments use the same fixed random seed and deterministic training protocol. Differences of less than one percentage point are interpreted as numerical trends rather than evidence of statistical significance.

Under the IR-protected setting, zero-initialized residual mapping achieves 44.380% mAP@0.5, whereas normally initialized residual mapping and full fine-tuning of the protected branch obtain 43.645% and 43.967%, respectively. Zero initialization ensures that the auxiliary residual is initially zero, such that the injected feature remains identical to the original protected representation at the beginning of training. The auxiliary correction is then learned progressively during optimization. Because the differences among these configurations are smaller than one percentage point, they are interpreted as numerical trends consistent with zero-initialized residual learning rather than as evidence of statistical significance.

The injection-location experiments show that injecting auxiliary information only at the stem, P3, P4, or P5 stage produces 42.717%, 43.691%, 42.825%, and 42.471% mAP@0.5, respectively. The P3 + P4 and P3 + P4 + P5 configurations achieve 42.660% and 42.332%, respectively. All of these results are lower than the 44.380% obtained when zero-initialized residual injection is applied jointly at the stem, P3, P4, and P5 stages. These results indicate that the contribution of the auxiliary modality is not concentrated at a single feature level. Within the current architecture, multi-stage controlled injection is more effective for jointly exploiting shallow spatial information and high-level semantic information. The decrease to 42.332% after removing stem-level injection from the P3 + P4 + P5 configuration further suggests that early auxiliary information is an important part of the complete controlled feature flow.

The comparison of fusion operators further demonstrates the role of zero-initialized residual mapping. Direct addition and concatenation followed by a 1 × 1 convolution achieve 43.485% and 43.448% mAP@0.5, respectively, both lower than the 44.380% obtained by the zero-initialized residual configuration. Direct addition does not constrain the initial influence of auxiliary features, whereas concatenation-based fusion cannot guarantee that the initial output remains identical to the protected branch.

The module-replacement experiments evaluate the customized components within the complete model. Replacing MGFR with CBAM yields 48.608% mAP@0.5, compared with 49.530% for the complete model. Replacing SBGA with a conventional PAN–FPN reduces mAP@0.5 to 46.760%. Replacing HLFDE with a standard C3k2 block produces 47.523% mAP@0.5.

#### 4.3.2. Modality Degradation and Spatial Misalignment

Controlled degradations were applied only during evaluation, while the trained checkpoint and target annotations remained unchanged. Gaussian blur used kernel sizes of 3, 5, and 9. Additive Gaussian noise was applied with standard deviations of 10, 20, and 30 on the [0, 255] intensity scale. RGB illumination degradation was generated using factors of 0.7, 0.4, and 0.2. Simulated absence of the modality was implemented by replacing the corresponding input with a zero tensor. IR thermal saturation was generated using I’_IR_(x,y) = min(255, g I_IR_(x,y)), with gain coefficients 1.31, 1.675, and 2.07 for mild, medium, and severe saturation, respectively. The RGB image, image size, and target annotations were kept unchanged. For spatial misalignment, the IR image and target annotations were kept unchanged, while only the auxiliary RGB image was shifted horizontally to the right by 5, 10, or 20 pixels. Pixels that exceeded the right edge of the image were cropped, and the newly exposed region on the left was filled with zero-valued pixels. Circular wrapping was not used. Because IR is the protected modality in the MDCFT configuration, shifting the protected IR branch was not used as the original spatial-offset protocol. The corresponding robustness results are reported in Table 4.

For the auxiliary RGB modality, performance decreases progressively as blur, noise, and illumination degradation worsen. The mAP@0.5 values decrease from 48.79% to 44.48% across mild-to-severe blur, from 48.54% to 43.34% across Gaussian-noise levels, and from 48.94% to 42.40% across illumination factors. These results indicate limited tolerance to mild auxiliary RGB degradation.

For the protected IR modality, increasing the Gaussian-noise severity reduces mAP@0.5 from 47.65% to 31.80%, while increasing the thermal-saturation severity reduces it from 48.00% to 27.84%. Severe degradation of the protected IR modality causes a larger performance reduction than severe degradation of the auxiliary RGB modality.

In the absence of the simulated modality, replacing the auxiliary RGB modality with a zero tensor retains 42.57% mAP@0.5, whereas replacing the protected IR modality reduces mAP@0.5 to 4.93%. Cross-modal spatial misalignment of 5, 10, and 20 pixels yields mAP@0.5 scores of 46.91%, 41.98%, and 30.14%, respectively. The model, therefore, has limited tolerance for the absence of auxiliary RGB and mild cross-modal spatial misalignment, but it does not replace geometric registration or online reliability estimation.

#### 4.3.3. Scale- and Condition-Stratified Evaluation

To analyze detection performance across target scales, COCO-style area thresholds are adopted at an input resolution of 640 × 640. Small objects have areas below 32^2^ pixels, medium objects have areas between 32^2^ and 96^2^ pixels, and large objects have areas above 96^2^ pixels. The scale-stratified results are reported in Table 5.

Compared with the MDCFT baseline AP_S_ of 15.042%, the complete model improves the AP_S_ to 16.919%. However, replacing HLFDE with C3k2 yields an APS of 17.492%. Therefore, the scale-stratified results support the improvement of the complete hierarchical feature flow over the MDCFT baseline but do not establish that HLFDE is independently optimal for small-object AP.

The evaluation set was partitioned into mutually exclusive illumination, occlusion, and density subsets using deterministic operational rules because the dataset does not provide native labels for these conditions. For illumination grouping, the 25th-percentile intensity of the RGB grayscale image and the fraction of IR pixels with intensity below 64 were computed for each image. These features were converted to validation-set percentile ranks, and the daytime score was defined as S_day_ = 0.70 R(P25_RGB_) + 0.30 R_inverse_(f_IR_ < 64). Images were sorted in descending order; the first 742 images were assigned to day and the remaining 483 to night, with the filename order used to resolve ties. The dataset does not provide native human-annotated occlusion labels. The occlusion-proxy score was defined as S_occ_ = 0.25 R(N_obj_) + 0.75 R_inverse_(mean box-area ratio), where N_obj_ is the number of annotated objects. The top 313 images were assigned to the occluded-proxy subset, and the remaining 912 to the non-occluded-proxy subset. For density grouping, the score was defined as S_dense_ = 0.25 R(O_max_) + 0.75 R_inverse_(mean box-area ratio), where O_max_ is the maximum pairwise intersection-over-smaller-box-area ratio. The top 541 images were assigned to the dense/crowd-proxy subset, and the remaining 684 to the sparse-proxy subset. These subsets are operational proxies rather than human-annotated ground-truth labels; the exact image lists are provided as supplementary manifests. The resulting condition-stratified performance is summarized in Table 6.

Overall, the additional experiments provide three findings. First, the three-seed verification shows that IR-only and IR-protected MDCFT remain numerically close, whereas MDCFT-HSM consistently yields higher results. Second, the evaluation-time degradation experiments show limited tolerance to mild degradation or the absence of the auxiliary RGB modality, but a substantially larger performance loss under severe degradation or the absence of the protected IR modality. Under the specified thermal-saturation protocol, the verification mAP@0.5 values for mild, medium, and severe saturation were 47.995%, 40.516%, and 27.836%, respectively. Third, the condition-stratified analysis shows lower performance on the occlusion-proxy and dense/crowd-proxy subsets than on their corresponding complementary subsets. These results support the training stability and controlled robustness of MDCFT-HSM, but do not establish online sample-level reliability adaptation or universal robustness to arbitrary sensor failures.

The day and night subsets achieve mAP@0.5 scores of 48.820% and 50.621%, respectively. Although the night subset yields a slightly higher result, this difference is influenced by the composition of the current subsets, the target distributions, and the sensor imaging conditions. It should, therefore, not be interpreted as evidence that nighttime scenes are generally easier than daytime scenes. The non-occluded and occluded subsets achieve 51.640% and 43.382% mAP@0.5, respectively, corresponding to a decrease of 8.258 percentage points. The sparse and dense/crowd subsets achieve 51.480% and 47.065%, respectively, corresponding to a decrease of 4.415 percentage points. Compared with illumination variation, occlusion, and high target density cause more substantial performance degradation, indicating that missing object boundaries, overlapping responses between adjacent targets, and complex local backgrounds remain the principal difficulties for the current model.

### 4.4. Comparison on RGBTDronePerson

Table 7 compares representative RGB-T detection methods on RGBTDronePerson, with metrics including Crowd, Person, Rider, mAP@0.5, FPS, Params, and GFLOPs.

Based on the reported values in Table 7, MDCFT-HSM achieves 49.5% mAP@0.5, matching DPDETR and remaining 1.4 percentage points below LF-DETR. MDCFT-HSM uses 7.38 M parameters, fewer than DPDETR and LF-DETR, although its computational cost is not the lowest among all of the compared methods. These cross-paper results position MDCFT-HSM as a competitive option in the accuracy–complexity trade-off rather than establishing universal superiority.

FPS values are listed for completeness but are not used for direct ranking because the reported hardware, inference frameworks, precision modes, and post-processing pipelines differ.

### 4.5. Additional Evaluation on DroneVehicle

DroneVehicle is used to observe the behavior of MDCFT-HSM in a vehicle-oriented UAV RGB-T scenario. Compared with the person, rider, and crowd categories in RGBTDronePerson, vehicles have different appearances, thermal responses, and background relations.

MDCFT-HSM achieves 83.60% mAP@0.5 and 63.28% mAP@0.5:0.95 on DroneVehicle. Its reported mAP@0.5 is higher than that of SuperYOLO and MFPT, but lower than that of HyperDet-N and HyperDet-S. Because the competing results were obtained under different implementations, Table 8 is used to contextualize the cross-dataset performance of MDCFT-HSM rather than establish a unified efficiency ranking.

### 4.6. Visualization Analysis

**Figure 8:**
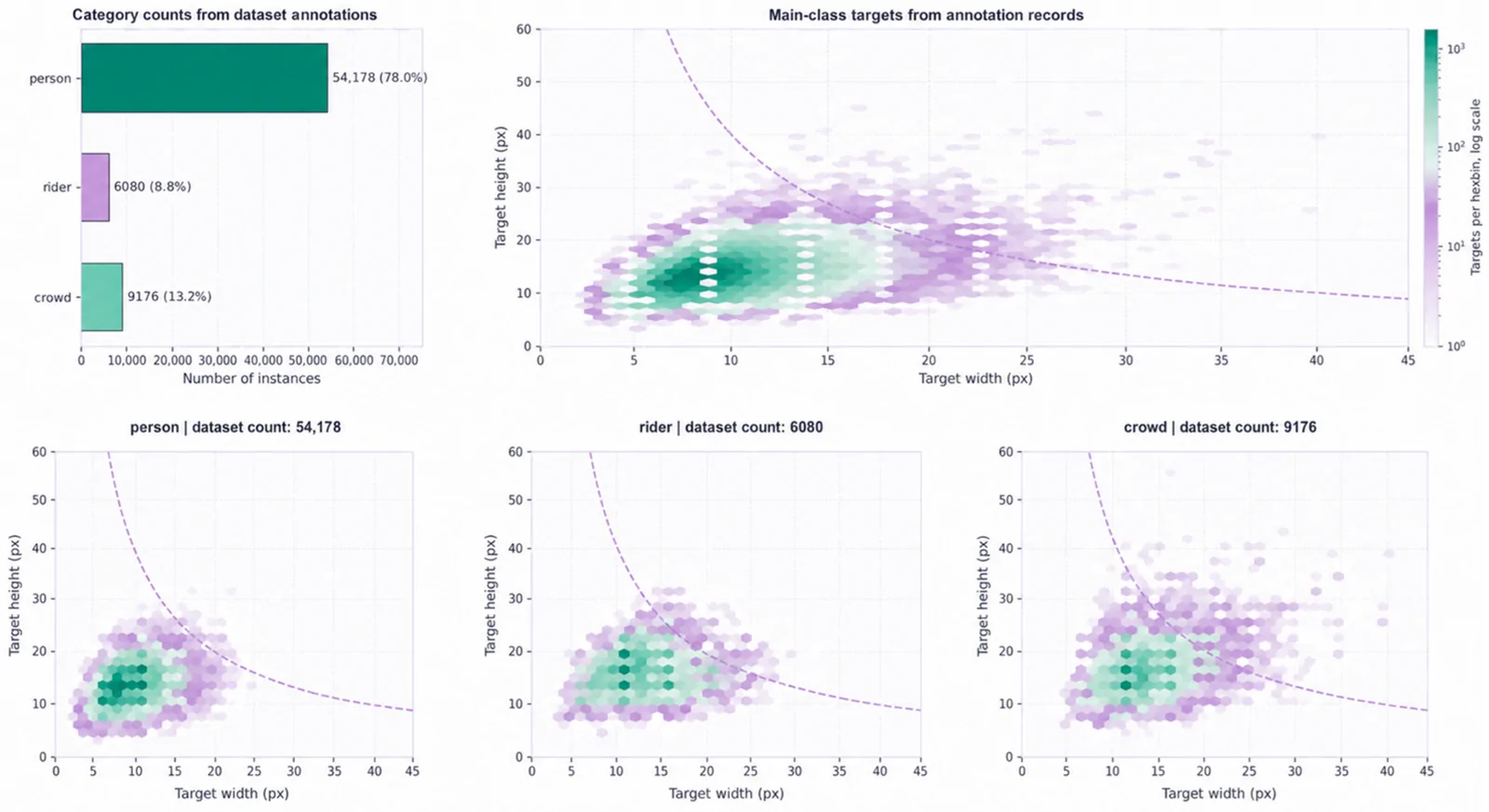
Target scale and category distribution on RGBTDronePerson. The purple dashed curves indicate a constant target bounding-box area of 400 pixels^2^ (width × height), where width and height correspond to the horizontal and vertical axes, respectively. This reference is used only to visualize the target-size distribution and is not the COCO-style scale threshold adopted in Section 4.3.3.

**Figure 9:**
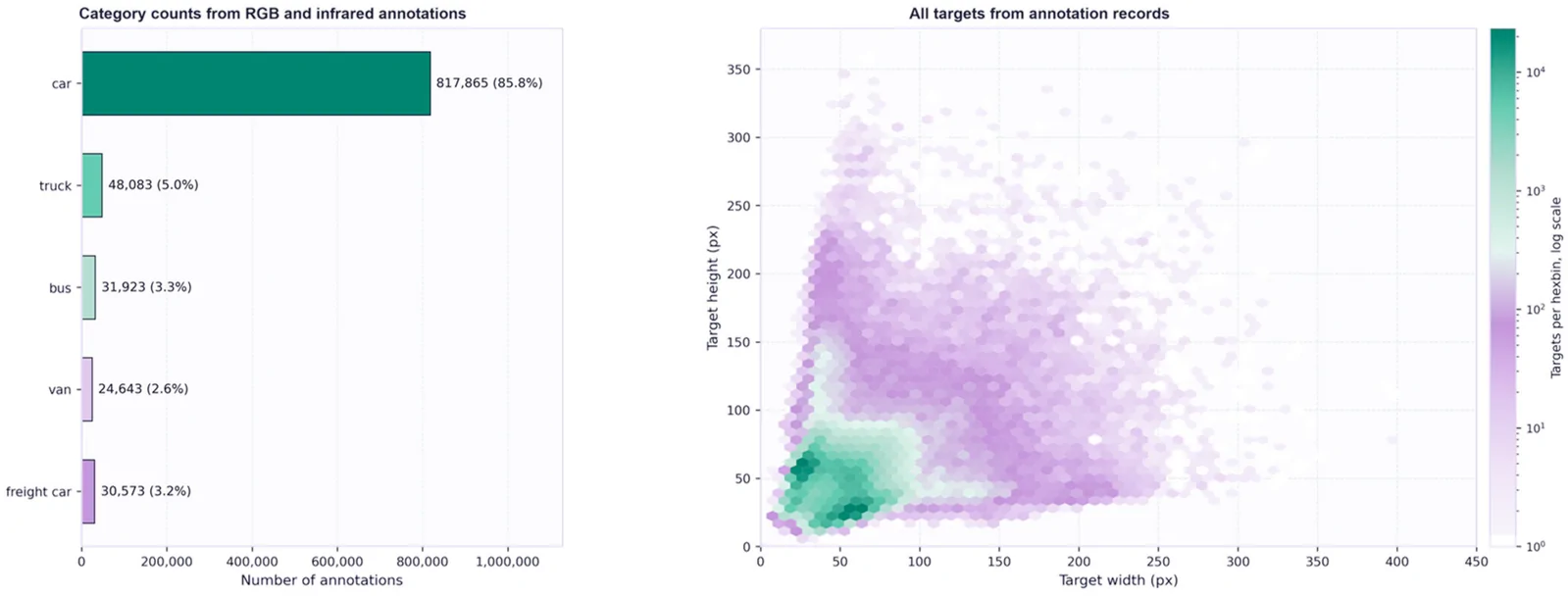
Target scale and category distribution on DroneVehicle.

The visualizations provide qualitative context for the dataset distributions, convergence behavior, and activation responses observed in the quantitative experiments. Figure 8 and Figure 9 show the target-scale and category distributions of RGBTDronePerson and DroneVehicle.

**Figure 10:**
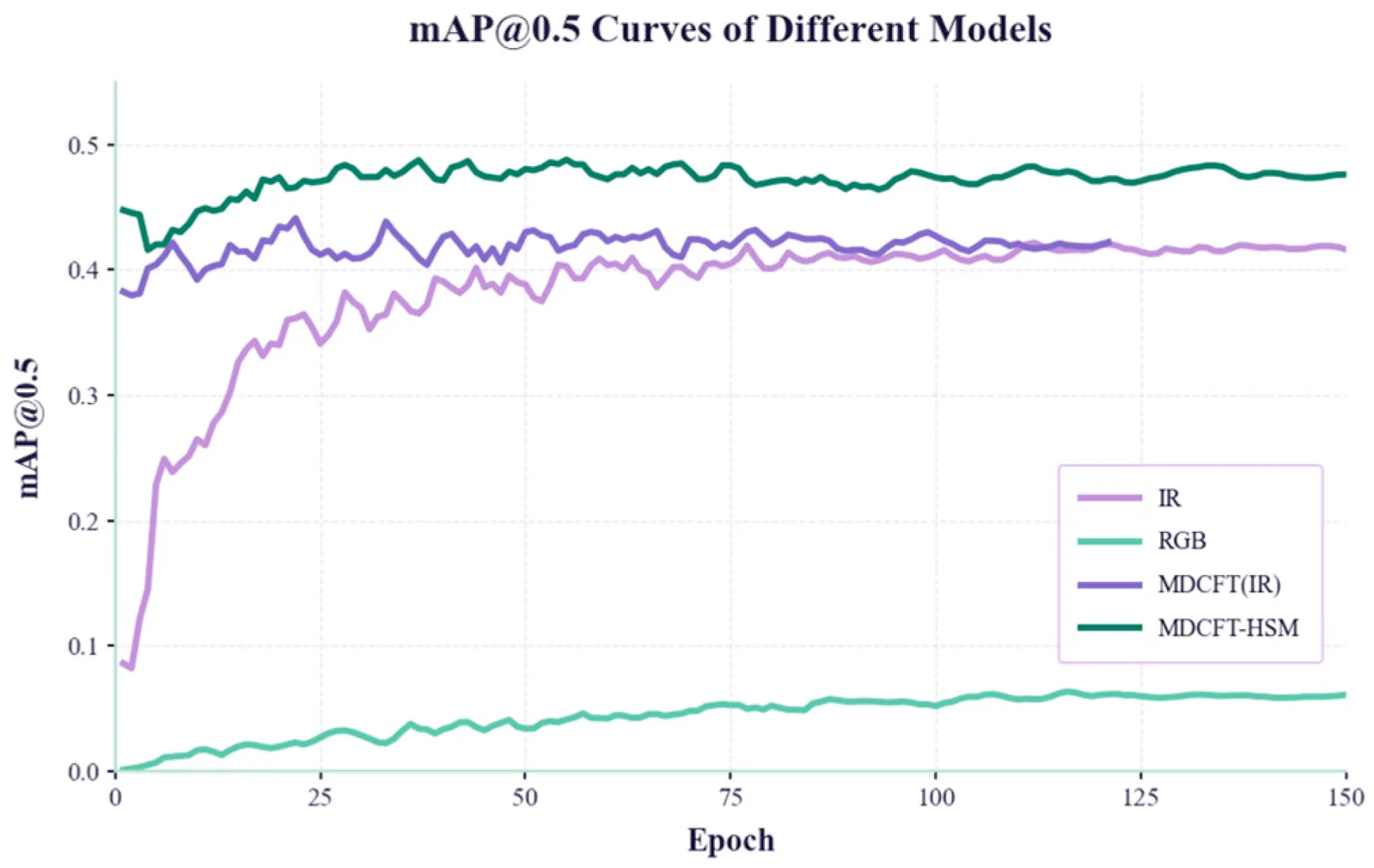
Training curves of mAP@0.5 for representative model variants.

Figure 10 shows the mAP@0.5 training curves of representative model variants. The RGB-only model converges to a substantially lower level than the IR-only model, confirming that the low RGB-only result is not caused by a failure to start training. MDCFT (IR-protected) remains close to the IR-only baseline, whereas the complete MDCFT-HSM reaches a higher value during later training. The curves are used to examine convergence behavior and are not treated as quantitative evidence of localization.

**Figure 11:**
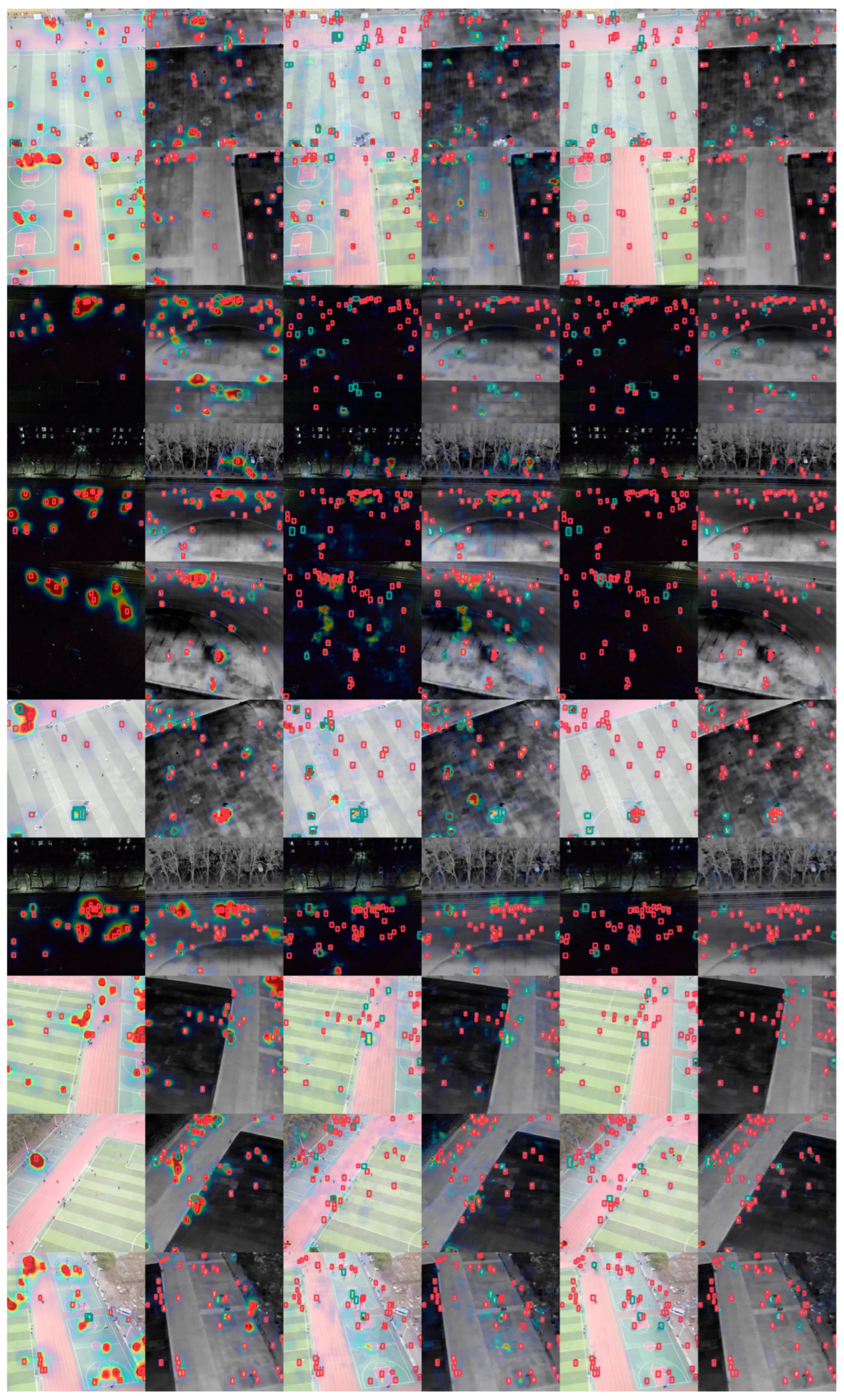
Detection heatmap comparison among RGB-only, IR-only, MDCFT, and MDCFT-HSM on ten representative paired RGB-T samples. From left to right, the columns show RGB-only on RGB input, IR-only on IR input, MDCFT on RGB input, MDCFT on IR input, MDCFT-HSM on RGB input, and MDCFT-HSM on IR input. Each row uses the same crop and activation-normalization scale. Red rectangles indicate ground-truth annotations, green/cyan rectangles indicate model-predicted bounding boxes, and the heatmap colors represent normalized activation intensity; warmer colors (yellow, orange, and red) indicate stronger responses, whereas cooler colors (blue and cyan) indicate weaker responses.

Figure 11 provides a qualitative comparison of the activation responses of the RGB-only, IR-only, MDCFT, and MDCFT-HSM models. In several illustrated samples, MDCFT-HSM produces more compact activation responses around multiple small-object regions, whereas the single-modality baselines show weaker or more spatially diffuse responses. The MDCFT and MDCFT-HSM results also indicate that controlled auxiliary-feature injection and the subsequent hierarchical modeling can refine the spatial concentration of responses in dense and low-contrast scenes. These visualizations are used only as qualitative evidence and do not replace the quantitative results reported in Table 2, Table 5 and Table 6.

### 4.7. Detection Performance Evaluation with Three Random Seeds

To assess training variability, the principal baseline and complete-model experiments were repeated using random seeds of zero, one, and two. The sample mean and standard deviation are reported in Table 9. The IR-only baseline and IR-protected MDCFT remain very close across the three seeds, whereas MDCFT-HSM consistently achieves a higher mAP@0.5. Differences below one percentage point are, therefore, interpreted as numerical trends rather than statistically established superiority.

## 5. Discussion

The experimental results demonstrate that MDCFT primarily stabilizes the IR-protected baseline, while the substantial accuracy improvements are attributed to the orderly integration of HLFDE, SBGA, and MGFR. The full proposed model elevates the mAP@0.5 metric from 44.38% (IR-protected MDCFT baseline) to 49.53%. Scale-stratified evaluation results further validate that our method yields superior APS performance compared with the MDCFT baseline, even though the C3k2 replacement module achieves a marginally higher APS score than the complete model. Collectively, these findings verify the effectiveness of the full hierarchical feature flow architecture. However, the results also indicate that each customized module does not deliver optimal performance independently across all target scales.

The distinction of MDCFT-HSM lies in its ordered treatment of auxiliary-feature entry, shallow-detail preservation, cross-level transfer, and feature recalibration before the Detect head. CFT [5], YOLOFusion [6], ICAFusion [7], and DPDETR [8] emphasize cross-modal interaction structures, whereas DHANet [15], multibranch progressive fusion [16], and prior-enhanced mixture-of-experts fusion [17] emphasize hierarchical interaction or routing selection. MDCFT-HSM combines a frozen protected branch with zero-initialized residual injection and staged small-object feature modeling.

This study has several notable limitations that warrant acknowledgment. First, the protected branch is determined based on single-modality evidence at the dataset level and is kept fixed throughout the inference stage. Results from the RGB-protected MDCFT analysis indicate that this selection strategy is purely heuristic and cannot guarantee an optimal feature propagation direction for multimodal fusion. Furthermore, a full evaluation of the complete RGB-protected MDCFT-HSM configuration is not included in this work. Second, the subsets corresponding to day/night scenarios, object occlusion, and crowd density are deterministically generated operational proxies derived from image statistics and bounding box annotations, instead of relying on human-labeled ground-truth condition labels. As such, the associated results should be interpreted as conditional analyses specific to the proposed evaluation protocol rather than universally applicable findings. Third, the presented method lacks geometric registration procedures, as well as reliability estimation, at the sample, region, and temporal levels. Consequently, its performance degrades substantially in the presence of large RGB-IR spatial offsets or severe quality degradation, as well as when the protected IR modality is partially or completely missing. Finally, all robustness experiments are conducted with controlled perturbations during inference, which fails to fully characterize real-world sensor faults or simultaneous quality degradation of both input modalities in practical scenarios.

These inherent limitations and experimental findings provide valuable insights and methodological inspiration for interested readers and subsequent research. The performance gaps between single-modality and dual-modality input conditions indicate the need for adaptive modality fusion and dynamic feature scheduling under varying sensor conditions. Related YOLO-based research has also investigated task-specific feature enhancement for photovoltaic-cell defect detection [39], indicating the broader relevance of feature-oriented detection design beyond UAV RGB-T detection.

## 6. Conclusions

This paper proposes MDCFT-HSM for low-altitude UAV RGB-T small-object detection. MDCFT uses a frozen protected branch and zero-initialized auxiliary residual injection at the stem, P3, P4, and P5 stages. HLFDE preserves shallow high- and low-frequency evidence. SBGA performs gated cross-level detail transfer in the neck, and MGFR recalibrates the P3, P4, and P5 features before the Detect head. The ablation results show that these components provide complementary improvements when organized as an ordered feature flow.

Comprehensive quantitative experiments demonstrate a competitive accuracy–complexity trade-off for MDCFT-HSM on two UAV RGB-T detection benchmarks. On the RGBTDronePerson dataset, MDCFT-HSM achieves a mAP@0.5 of 49.53% and a mAP@0.5:0.95 of 18.97%, with a compact parameter size of 7.38 M, 44.2 GFLOPs, and a real-time inference speed of 60.0 FPS. For the DroneVehicle dataset, the model yields further improved detection results, reaching 83.60% mAP@0.5 and 63.28% mAP@0.5:0.95. Additional robustness evaluations reveal that the model shows limited tolerance to auxiliary RGB absence and mild cross-modal spatial misalignment. However, severe degradation or complete loss of the dominant thermal infrared modality leads to a dramatic drop in overall detection accuracy, which significantly limits the practical applicability of the model in complex low-altitude remote sensing scenarios.

To address the aforementioned limitations and further enhance the practicability and generalization capability of UAV RGB-T small-object detection systems, future research will concentrate on three core directions. First, sample-level modality reliability estimation mechanisms will be developed to dynamically quantify the quality and contribution of dual-modal data during inference. Second, registration-aware cross-modal fusion strategies will be explored to adaptively mitigate the negative impacts of spatial misalignment between visible-light and thermal infrared images. Third, specialized optimization schemes will be designed to strengthen the model robustness against simultaneous degradation or absence of both modalities, enabling more stable and reliable detection performance in extreme and complex field environments.

## Figures and Tables

**Table 1:** Training and evaluation configuration of MDCFT-HSM.

Setting	Value
Input size	640 × 640
Input channels	6 (3 RGB + 3 IR)
Input modality	Paired RGB-T
Epochs	150
Batch size	16
Optimizer	SGD
Learning rate	0.01
Momentum	0.937
Weight decay	0.0005
Mosaic/close mosaic	0.2/20
Training image pairs	4900
Evaluation image pairs	1225
Training annotations	54,973
Evaluation annotations	14,460
Split policy	Official training/evaluation split (4:1; 4900 training and 1225 evaluation pairs)
Operating system	Not recorded in the archived experimental record
Random seed	0
Deterministic training	Yes
CPU	Intel Core i9-13900K
Memory	32 GB RAM
GPU	NVIDIA GeForce RTX 4090, 24 GB
Python	3.8
PyTorch	1.13
CUDA	11.7
cuDNN	8.5
Ultralytics	8.3.75
IR initialization	Trained IR-only checkpoint
RGB initialization	YOLO11n pretrained weights
IR conversion	Single-channel IR replicated to three channels using Gray2BGR.
Haar decomposition	One-level Haar DWT/IDWT
Transformer depth	Two Transformer blocks per stage
P3/P4/P5 channels	64/128/256
Frozen components	IR backbone, neck, and Detect head
Trainable components	RGB branch, zero-initialized residual mappings, HLFDE, SBGA, and MGFR

**Table 2:** Ablation study on the RGBTDronePerson dataset.

Model	H	S	M	GFLOPs	Size (MB)	Precision	Recall	mAP@0.5 (%)
YOLOv11n (IR-only)				6.4	5.2	0.5294	0.4909	44.19
YOLOv11n (RGB-only)				6.4	5.2	0.1345	0.1326	5.95
MDCFT (IR-protected)				9.2	8.8	0.5551	0.4772	44.38
MDCFT (IR-protected)	√			39.7	11.9	0.5422	0.5012	47.37
MDCFT (IR-protected)	√		√	41.8	14.0	0.5503	0.4837	44.77
MDCFT (IR-protected)	√	√		42.1	12.5	0.5400	0.5043	48.17
MDCFT (IR-protected)			√	11.3	10.9	0.5662	0.4768	46.00
MDCFT (IR-protected)		√		11.6	9.4	0.5529	0.5013	46.04
MDCFT (IR-protected)		√	√	13.7	11.5	0.5540	0.4924	46.20
MDCFT-HSM	√	√	√	44.2	14.6	0.5516	0.5226	49.53

Note: H, S, and M denote HLFDE, SBGA, and MGFR, respectively.

**Table 3:** Design rationale and module ablation results of the MDCFT framework.

Configuration	Precision	Recall	mAP@0.5 (%)	mAP@0.5:0.95 (%)	Params (M)
IR-protected, zero-initialized residual mapping	0.5551	0.4772	44.380	16.909	4.427
Normally initialized residual mapping	0.5124	0.4570	43.645	16.974	4.427
Full fine-tuning of the protected branch	0.5241	0.4956	43.967	16.209	4.427
RGB-protected, IR auxiliary	0.5299	0.5018	45.896	17.281	4.427
Stem-only injection	0.5299	0.4599	42.717	16.272	4.427
P3-only injection	0.5266	0.4841	43.691	16.430	4.427
P4-only injection	0.5064	0.4861	42.825	15.937	4.427
P5-only injection	0.5227	0.4612	42.471	15.731	4.427
P3 + P4 injection	0.5091	0.4685	42.660	16.071	4.427
P3 + P4 + P5 injection without the stem	0.5255	0.4680	42.332	15.799	4.427
Direct Add fusion	0.5091	0.4675	43.485	16.601	3.542
Concatenation followed by a 1 × 1 convolution	0.5158	0.4827	43.448	15.976	3.739
C3k2 replacing HLFDE	0.5533	0.5233	47.523	18.742	5.812
CBAM replacing MGFR	0.5617	0.5238	48.608	18.337	6.585
PAN–FPN replacing SBGA	0.5482	0.5017	46.760	17.913	7.067
Complete MDCFT-HSM	0.5516	0.5226	49.530	18.970	7.380

**Table 4:** Results under modal degradation, simulated modality absence, and cross-modal spatial misalignment.

Test Condition	Precision	Recall	mAP@0.5 (%)	mAP@0.5:0.95 (%)
Clean paired input	0.5516	0.5226	49.530	18.970
RGB blur, mild (kernel size k = 3)	0.5444	0.5142	48.787	18.629
RGB blur, medium (kernel size k = 5)	0.5295	0.4980	47.351	18.022
RGB blur, severe (kernel size k = 9)	0.4992	0.4641	44.478	16.807
RGB Gaussian noise, σ = 10	0.5411	0.5111	48.539	18.534
RGB Gaussian noise, σ = 20	0.5229	0.4907	46.806	17.756
RGB Gaussian noise, σ = 30	0.4865	0.4526	43.339	16.276
RGB illumination factor 0.7	0.5455	0.5153	48.936	18.686
RGB illumination factor 0.4	0.5268	0.4944	47.153	17.889
RGB illumination factor 0.2	0.4788	0.4400	42.398	15.897
IR Gaussian noise, σ = 10	0.5340	0.5007	47.648	18.097
IR Gaussian noise, σ = 20	0.4843	0.4453	42.794	16.011
IR Gaussian noise, σ = 30	0.3668	0.3251	31.798	11.534
IR thermal saturation, mild	0.5373	0.5043	47.995	18.230
IR thermal saturation, medium	0.4606	0.4170	40.516	14.892
IR thermal saturation, severe	0.3243	0.2827	27.836	9.902
RGB absent (zero tensor)	0.5312	0.4865	42.570	16.120
IR absent (zero tensor)	0.1458	0.1292	4.930	1.810
RGB–IR spatial misalignment, 5 pixels	0.5279	0.4968	46.910	17.720
RGB–IR spatial misalignment, 10 pixels	0.4765	0.4472	41.980	15.560
RGB–IR spatial misalignment, 20 pixels	0.3618	0.3297	30.140	10.880

**Table 5:** Scale-stratified detection results.

Model	mAP@0.5 (%)	mAP@0.5:0.95 (%)	AP_S_ (%)	AP_M_ (%)	AP_L_ (%)
YOLOv11n (RGB-only)	5.950	2.213	1.958	2.700	3.585
YOLOv11n (IR-only)	44.190	17.062	15.139	21.855	30.268
MDCFT (IR-protected)	44.380	16.909	15.042	22.688	32.600
MDCFT-HSM	49.530	18.970	16.919	26.609	33.122
C3k2 replacing HLFDE	47.523	18.742	17.492	23.354	34.298
PAN–FPN replacing SBGA	46.760	17.913	16.801	24.503	32.279

**Table 6:** Condition-stratified evaluation results.

Partition	Condition	Image Pairs	mAP@0.5 (%)	mAP@0.5:0.95 (%)
Illumination	Day	742	48.820	18.440
Illumination	Night	483	50.621	19.784
Occlusion	Non-occluded proxy	912	51.640	19.860
Occlusion	Occluded proxy	313	43.382	16.377
Density	Sparse proxy	684	51.480	19.940
Density	Dense/crowd proxy	541	47.065	17.744

**Table 7:** Comparison with representative methods on the RGBTDronePerson dataset.

Method	Modality	Crowd AP@0.5	Person AP@0.5	Rider AP@0.5	mAP@0.5 (%)	FPS (Reported)	Params (M)	GFLOPs
QFDet [1]	RGB-T	29.9	46.1	50.3	42.1	5.7	60.3	81.4
CFT [5]	RGB-T	35.5	46.9	40.8	41.1	119.0	44.5	18.0
LF-DETR [18]	RGB-T	44.8	60.1	47.8	50.9	55.3	33.7	175.5
PTANet [9]	RGB-T	-	-	-	47.8	-	4.72	29.5
AODet [11]	RGB-T	-	-	-	47.7	-	-	-
ICAFusion [7]	RGB-T	37.8	45.3	44.7	42.6	28.4	23.3	14.9
DPDETR [8]	RGB-T	42.4	56.7	49.4	49.5	16.4	90.1	208.0
YOLOFusion [6]	RGB-T	33.8	52.2	48.0	44.7	36.1	16.3	-
VTsARNet [10]	RGB-T	-	-	-	40.4	10.5	6.9	-
SLBAF-Net [12]	RGB-T	36.3	44.2	30.3	36.9	69.0	11.0	29.9
DEYOLO [13]	RGB-T	37.3	51.7	38.8	42.6	91.1	22.9	61.7
DEGF-YOLO [14]	RGB-T	40.0	54.0	49.5	47.8	31.4	19.6	-
YOLOv11-RGBT [33]	RGB-T	33.2	38.9	33.4	35.1	106.4	4.4	9.0
RSDet [19]	RGB-T	-	-	-	44.0	50	9.5	25
M^2^D-LIF [20]	RGB-T	-	-	-	47.0	80	3.9	17.5
MDCFT-HSM	RGB-T	43.3	59.4	45.8	49.5	60.0	7.38	44.2

Note: “-” indicates that the corresponding metric was not reported. Results for competing methods were taken from the corresponding publications and were not re-evaluated under a unified hardware and software environment. The FPS of MDCFT-HSM was measured using the protocol described in Section 4.1, whereas the other FPS values are reported values from the original papers.

**Table 8:** Comparison with representative methods on the DroneVehicle dataset.

Method	Car AP@0.5	Freight Car AP@0.5	Truck AP@0.5	Bus AP@0.5	Van AP@0.5	mAP@0.5	mAP@0.5:0.95	Params (M)	GFLOPs	FPS (Reported)
YOLOv11n (RGB-only)	88.5	62.3	69.1	89.6	59.5	73.8	52.1	2.6	6.3	428.9
YOLOv11n (IR-only)	93.2	69.5	74.8	93.1	65.4	80.2	59.6	2.6	6.3	-
ICAFusion [7]	96.8	61.1	74.4	95.3	49.5	75.4	52.6	120.2	191.6	-
YOLOv11n-B3 [32]	96.8	73.5	79.2	95.5	69.5	82.9	62.5	2.7	8.1	358.1
HyperDet-N [32]	98.6	80.9	84.0	97.5	70.9	86.4	65.9	3.2	9.1	308.3
HyperDet-S [32]	98.7	80.9	86.0	97.8	73.9	87.5	67.1	11.7	32.4	242.1
SuperYOLO [34]	97.7	79.0	66.3	96.6	67.6	81.4	58.6	4.8	55.9	89.4
MFPT [35]	97.3	72.2	77.2	96.6	66.7	82.0	60.7	47.7	34.6	-
C^2^Former [36]	90.2	64.4	68.3	89.8	58.5	74.2	-	89.9	100.8	-
GM-DETR [37]	92.4	75.3	80.8	90.8	64.9	80.8	55.9	70.0	176.0	45.6
WaveMamba [38]	95.0	68.5	80.4	90.6	64.5	79.8	60.5	69.1	-	-
MDCFT-HSM	98.1	70.0	81.4	96.7	71.8	83.6	63.28	7.38	44.2	57.24

Note: “-” indicates that the corresponding metric was not reported. The class-wise values for car, freight car, truck, bus, and van are AP@0.5.

**Table 9:** Three-seed verification results on the RGBTDronePerson dataset.

Configuration	Seed 0	Seed 1	Seed 2	Mean ± SD (pp)
YOLOv11n, RGB-only	5.950	5.640	6.120	5.903 ± 0.243
YOLOv11n, IR-only	44.190	44.010	44.490	44.230 ± 0.242
MDCFT, IR-protected	44.380	44.110	44.590	44.360 ± 0.241
MDCFT-HSM	49.530	49.310	49.880	49.573 ± 0.287

## Data Availability

The public RGBTDronePerson and DroneVehicle datasets were used in this study. The processed dataset splits, model configurations, and evaluation scripts are available from the corresponding author on reasonable request.
